# Intermittent Neuralgia

**Published:** 1889-08

**Authors:** 


Intermittent neuralgia of the plexus brachialis dext., especially in shoulder and upper arm, caused by a concussion of the shoulder, attacks at a certain hour at night, regularly returning, with stitching pains, followed by weakness and stiffness; not free from pain in daytime. Arnica$^3$ cured the case at once, after having been endured for seventeen years. On account of the intermittency he also took one dose of Arseniate of Quinine, fourth dec., at night. (We do not think that this alternation was necessary; though Arnica has not intermittency, it has aggravation at night, and therefore claim the cure for the Arnica.) S. L.

